# Somatic symptom distress and ICD-11 prolonged grief in a large intercultural sample

**DOI:** 10.1080/20008066.2023.2254584

**Published:** 2023-09-28

**Authors:** Severin Hennemann, Clare Killikelly, Philip Hyland, Andreas Maercker, Michael Witthöft

**Affiliations:** a Department of Clinical Psychology, Psychotherapy and Experimental Psychopathology, Johannes Gutenberg University Mainz, Mainz, Germany; b Department of Psychology, University of Zurich, Zurich, Switzerland; c Department of Psychology, Maynooth University, Kildare, Ireland

**Keywords:** Grief, prolonged grief disorder, ICD-11, cross-cultural, somatic symptom distress, somatization, Trastorno de duelo prolongado, CIE-11, transcultural, malestar por síntomas somáticos, somatización, 哀伤, 延长哀伤障碍, ICD-11, 跨文化, 躯体症状障碍, 躯体

## Abstract

**Background:** Grief is a multi-faceted experience including emotional, social, and physical reactions. Research in ICD-11 prolonged grief disorder (PGD) in different cultural contexts has revealed different or potentially missing grief symptoms that may be relevant.

**Objective:** This study thus aimed to explore the prevalence of somatic symptom distress and its associations with grief and negative affect in a culturally diverse sample of bereaved individuals with symptoms of PGD.

**Methods:** Based on cross-sectional survey data from the Measurement and Assessment of Grief (MAGIC) project, this study included 1337 participants (mean age 23.79 yrs, 76.1% female) from three regions (USA: 62.3%, Turkey/Iran: 24.2%, Cyprus/Greece: 13.5%), who experienced a loss of a significant other. Associations between somatic symptom distress (Somatic Symptom Scale, SSS-8), symptoms of PGD (International Prolonged Grief Disorder Scale, IPGDS-33), anxiety (Generalized Anxiety Disorder Questionnaire, GAD-7), depression (Patient Health Questionnaire, PHQ-9) as well as demographic and loss related characteristics were investigated. Three hundred and thirteen participants (23.4%) scored above the proposed cut-off for clinically severe PGD.

**Results:** ‘High’ or ‘very high’ levels of somatic symptom distress were more frequent in a possible PGD group (58.2%), than in a non-PGD group (22.4%), *p* < .001, as divided per cut-off in the IPGDS. In a multiple regression analysis, PGD symptoms were significantly but weakly associated with somatic symptom distress (*β *= 0.08, *p* < .001) beyond demographics, loss-related variables, and negative affect. Negative affect (anxiety and depression) mediated the relationship of PGD symptoms with somatic symptom distress and the indirect effect explained 58% of the variance.

**Conclusions:** High levels of somatic symptom distress can be observed in a substantial proportion of bereaved across cultures. Our findings suggest that PGD is related to somatic symptom distress partly and indirectly through facets of negative affect.

## HIGHLIGHTS

- 30.8% of bereaved adults showed ‘high’ or ‘very high’ levels of somatic symptom distress.
- Anxiety and depression partially mediate relationship of PGD symptoms with somatic symptom distress.
- Findings encourage practitioners to consider somatic symptom distress in psychotherapeutic treatment of PGD.

## Abstract (es)

**Antecedentes:** El duelo es una experiencia multifacética que incluye reacciones emocionales, sociales y físicas. La investigación en el trastorno por duelo prolongado según la CIE-11(PGD en sus siglas en ingles) en diferentes contextos culturales ha revelado síntomas de duelo diferentes o potencialmente faltantes que pueden ser relevantes.

**Objetivo:** Este estudio tuvo como objetivo explorar la prevalencia del malestar por síntomas somáticos y su asociación con duelo y afecto negativo en una muestra culturalmente diversa de personas en duelo con síntomas de PGD.

**Métodos:** Basados en datos de encuestas transversales del proyecto de Medición y E*va*luación del Duelo (MAGIC), este estudio incluyó a 1.337 participantes (edad media 23.79 años, 76.1% mujeres) de tres regiones (EEUU: 62.3%, Turquía/Irán: 24.2%, Chipre/Grecia: 13.5%), que experimentaron la pérdida de una persona significativa. Se investigaron las asociaciones entre el malestar por síntomas somáticos (Escala de Síntomas Somáticos, SSS-8), síntomas de PGD (Escala Internacional de Trastorno de Duelo Prolongado, IPGDS), ansiedad (Cuestionario de Trastorno de Ansiedad Generalizada, GAD-7), depresión (Cuestionario de Salud del Paciente, PHQ-9), así como características demográficas y relacionadas con la pérdida. Trescientos trece participantes (23.4%) obtuvieron puntuaciones sobre el punto de corte propuesto para PGD clínicamente grave.

**Resultados:** Los niveles de malestar por síntomas somáticos ‘altos’ o ‘muy altos’ fueron más frecuentes en el grupo de posible PGD (58.2%), que en el grupo sin PGD (22.5%), *p* < .001, dividido por punto de corte en el IPGDS-33. En un análisis de regresión múltiple, los síntomas PGD se asociaron significativamente, pero débilmente con malestar por síntomas somáticos (*β* = 0.08, *p* < .001) más allá de las variables demográficas, relacionadas con la perdida y el afecto negativo. El afecto negativo (ansiedad y depresión) mediaron la relación de los síntomas PGD con el malestar por síntomas somáticos y el efecto indirecto explico el 58% de la varianza.

**Conclusiones:** Se puede observar altos niveles de malestar por síntomas somáticos en una proporción importante de personas en duelo en todas las culturas. Nuestros hallazgos sugieren que el PGD está relacionado con malestar por síntomas somáticos en parte e indirectamente a través de facetas del afecto negativo.

## Abstract (zh)

**背景：**哀伤是一种多方面的体验，包括情绪、社会和身体反应。在不同文化背景下对ICD-11延长哀伤障碍（PGD）的研究揭示了可能相关的不同或可能缺失的哀伤症状。

**目的：**因此本研究旨在一个具有文化多样性的具有PGD症状的丧亲者样本中探讨躯体症状障碍的流行率及其与哀伤和负性情感的关联。

**方法：**基于哀伤测量和评估（MAGIC）项目的横断面调查数据，本研究包括来自三个地区（美国：62.3%，土耳其/伊朗：24.2%，塞浦路斯/希腊：13.5%）的1337名参与者（平均年龄23.79岁，76.1%女性），他们经历了丧失另一半。调查了躯体症状障碍（躯体症状量表，SSS-8）、PGD症状（国际延长哀伤障碍量表，IPGDS）、焦虑（广泛性焦虑症问卷，GAD-7）、抑郁（患者健康问卷，PHQ-9）以及人口统计学和丧失相关特征之间的关联。313名受试者（23.4%）的得分高于临床严重PGD的拟议临界值。

**结果：**以IGPDS-33中临界值划分，‘高’或‘非常高’水平的躯体症状障碍在可能的PGD组（58.2%）比在非PGD组（22.4%）更常见，*p* < .001。 在多元回归分析中，PGD症状与躯体症状障碍（*β* = 0.08，*p* < .001）的相关性显著但弱，控制了人口统计学、丧失相关变量和负性情感。负性情感（焦虑和抑郁）中介了PGD症状与躯体症状障碍的关系，间接效应解释了58%的方差。

**结论：**在不同文化中，在相当大比例的失去亲人者中可以观察到高水平的躯体症状障碍。我们的研究结果表明，PGD通过负性情感的各个方面部分和间接地与躯体症状障碍有关。

## 1. Introduction

After the death of a loved one, grief is a natural process that includes emotional, social, or physical reactions, which usually subside over time. However, some individuals may experience ongoing symptoms of grief that are highly distressing and impairing, as described by the prolonged grief disorder (PGD). Recently, a representative sample study from Germany found prevalence rates of 3.3% and 4.2% in bereaved, depending on the diagnostic system (Rosner et al., 2021), while the risk for developing PGD can increase up to 49% after unnatural losses according to a systematic review (Djelantik et al., 2020). PGD is a new mental health disorder recognized in the 11th edition of the International Classifications of Diseases (ICD-11) (Killikelly & Maercker, 2017) and recently included in the Diagnostic Statistical Manual version 5 text revision (DSM 5 TR) (Prigerson et al., 2021). In the ICD-11 and DSM 5 TR PGD is defined by core symptoms of pervasive and persistent longing for the deceased or preoccupation with the deceased that is accompanied by feelings of intense emotional pain. Additionally, these symptoms must persist for an extended period of time (at least six months following the bereavement or at least 12 months following bereavement for the DSM 5 TR), should exceed normal cultural or religious expectations, and cause significant impairment in daily functioning. The cultural caveat is a new addition to the ICD-11 disorder definition and states that symptoms of disordered grief must exceed the expected cultural norms for the duration, intensity, and severity of symptoms. However, currently, the cultural norms for grief symptoms have not been systematically documented making it difficult for clinicians to include the wider cultural context in their diagnostic decision-making. Recently, research exploring ICD-11 PGD grief symptoms in different cultural contexts has revealed different or potentially missing grief symptoms that may be relevant. For example, semi-structured interviews with healthcare workers in China, Japan, and Switzerland, as well as bereaved Syrian refugees, found that somatic symptoms were identified as important indicators of grief, and these symptoms are currently absent from the ICD-11 PGD definition (Killikelly et al., 2020; Stelzer et al., 2020).

### 1.1. Grief and somatic symptom distress

Numerous studies have documented an increased prevalence of somatic symptom distress (also referred to as somatization) and associated somatoform disorders in various groups of bereaved individuals, including refugees (Steil et al., 2019), widows (Kim et al., 2017; Morina & Emmelkamp, 2012), parents who lost a child (Zhou et al., 2020), or survivors of natural disasters (Kristensen et al., 2012). A representative survey in the general Hungarian population found that 15% (males) to 27% (females) of bereaved adults showed clinically relevant levels of somatic symptom distress (Thege et al., 2012). Moreover, in a clinical trial investigating cognitive–behavioral therapy (CBT) for prolonged grief, 54% of outpatients were diagnosed with a comorbid somatoform disorder (Rosner et al., 2014). Previous work with Cambodian refugees has also revealed the importance of somatic symptoms associated with both bereavement and trauma (Hinton et al., 2013). Intrusive memories or recall of the deceased was found to increase somatic symptoms and distress; these were then attributed to a culturally specific symptom ‘khyàl attack’ which is translated as a ‘wind attack’. These are somatic symptoms caused by a disruption of ‘khyal’ or the wind that flows alongside blood in the body. This ‘wind attack’ is highly prevalent and predictive of distress in Cambodian refugees. Furthermore, ‘Ulysses syndrome’ was described as symptoms such as nervousness, migraine, insomnia, and headaches arising from the stress of forced migration (Bianucci et al., 2017). These findings highlight the importance of considering culturally relevant symptoms of distress beyond the standard definitions in the ICD-11 of grief, and the importance of somatic symptoms, in particular.

### 1.2. Relationship between stress-related disorders and somatic symptom distress

Previous research has also confirmed the importance of somatic symptom distress in predicting depression severity and stress-related disorders such as posttraumatic stress disorder (PTSD) (Jongedijk et al., 2020; Kocalevent et al., 2013; Löwe et al., 2008), demonstrating both commonalities and distinct profiles and mechanisms (Boelen & van den Bout, 2005; Rief et al., 2010). For example, a recent survey-based cohort study (Astill Wright et al., 2021) found a high prevalence of approximately 70% of somatic symptom distress in adult participants with complex PTSD, which may indicate shared vulnerability and maintaining biological and psychosocial factors, such as bodily hyperarousal, dysregulated stress response (e.g., in terms of altered hypothalamic–pituitary–adrenal axis function and hypercortisolism), reduced immune response, negative repetitive thinking and heightened self-attention (Brennstuhl et al., 2015; Ehring, 2021; McAndrew et al., 2019; Rief & Barsky, 2005). Furthermore, there is a strong body of work purporting the primacy of somatic experiences alongside depression in Asian cultures (Grover & Ghosh, 2014; Zhou et al., 2016). Interestingly, Ryder and Chentsova-Dutton (2012) present the idea that somatic symptom distress in Chinese culture could reflect a ‘cultural script’ for depression, in other words, a somatic form of distress or symptom communication may be more culturally acceptable and provide access to support. Research in bereaved east-European adults (Thege et al., 2012) suggests that somatic symptom distress is not a direct consequence of bereavement, but rather mediated by negative affect as a core component of anxiety and depression (Clark & Watson, 1991). While a mediating effect of anxiety was found for both genders, depression only mediated the relationship between bereavement and somatic symptoms in male bereaved, indicating a gender-sensitive influence. In line with the assumption that elevated somatic symptom distress is the consequence of negative affective states are experimental studies showing that the induction of negative affective states (and sadness in particular (Sauer & Witthöft, 2017), as well as the presentation of negative affective cues (e.g. pictures), can cause increases in somatic symptom distress (Bogaerts et al., 2010; van den Houte et al., 2019). In this current study, we have the opportunity to explore the relationship between grief severity and somatic symptom distress in three regions (USA, Cyprus/Greece, Turkey/Iran) as part of a large international collaboration (Killikelly et al., 2023). A key aim of this project is to include participants, contexts, and perspectives from the ‘majority world’ i.e. from participants of non-WEIRD countries (white, educated, industrialized, rich democratic nations) (Henrich et al., 2010). For example, previous cross-cultural research has confirmed that Greek participants may present a unique profile of somatic symptoms in the context of depression. When compared with Australian patients, Greek patients presented with more hyperventilation while Australians with more insomnia (Marmanidis et al., 1994). Interestingly, a psychometric validation study of the Beck Depression Inventory (BDI-II) in an Iranian sample found a two-factor model: an affective/cognitive factor and a separate somatic/vegetative factor not found in Western validations. This indicated the importance of somatic symptoms in the Iranian reporting of depression (Ghassemzadeh et al., 2005). In a sample of Turkish migrants accessing health support, all reported psychological distress when asked directly however, somatic symptoms were more frequently spontaneously reported as an explanation for distress (Gilgen et al., 2005).

### 1.3. Aims

The first objective of this study was to estimate the prevalence of self-reported somatic symptom distress and PGD symptoms in a large, cross-cultural sample who experienced the death of a significant other. Based on previous survey studies in various cultural samples of bereaved people, we expected a high level of somatic symptom distress, particularly in individuals with a high probability of PGD (Kristensen et al., 2012; Thege et al., 2012; Zhou et al., 2020). Secondly, we explored the relationship between somatic symptom distress and PGD symptoms, which would have implications for a shared vulnerability (Brennstuhl et al., 2015; McAndrew et al., 2019) and the role of somatic symptom distress in PGD diagnosis (Stelzer et al., 2020). Therein, we assumed both a higher prevalence of somatic symptom distress and a stronger association with PGD symptoms in females compared to males (Thege et al., 2012). Thirdly, based on previous surveys and experimental studies (Bogaerts et al., 2010; Grover & Ghosh, 2014; Sauer & Witthöft, 2017; Thege et al., 2012) we expected a mediating role of negative affect (i.e. depression, anxiety), which would implicate that underlying symptoms of depression or anxiety can be ‘masked’ by somatic symptom distress in bereaved people to some extent.

## 2. Methods

### 2.1. Design and procedure

This cross-sectional, survey-based study is part of a large research collaboration (MAGIC, Measurement and Assessment of Grief project) to study grief symptoms in international cross-cultural samples led by the University of Arizona, Network for International Collaborative Exchange (NICE) (for more information on NICE see: Cuccolo et al., 2021). The data collected for the MAGIC project is open access (https://osf.io/tyz3u/) and can be used to conduct different analysis or re-analysis of the current data set. The original published report conducted a psychometric validation of the IPGDS scale and a network analysis to examine core symptoms of PGD in different cultural contexts (see Killikelly et al., 2023). The data were collected at more than 23 universities across 5 countries between August 2019 and June 2020. Data were collected through social media platforms and institutional emailing lists in each country using snowball sampling. The following procedure was used in each country: a standard email that contains information on the study as well as a link to the online survey – created in Qualtrics and containing a consent form at the beginning of the survey was sent via weblink to local universities as well as local hospice and bereavement institutions (e.g. Hospice at Tucson Medical Center; Hospice of the Valley; TuNudito) with the request to forward this information to potentially eligible clients/patients. In addition, information about this study was provided on websites (e.g. Department of Psychology website), via university-wide list serves, and via campus advertisements and flyers. The online survey link and study coordinator’s contact information (email and phone) were included in all of the recruitment materials distributed so potential participants can access the online survey directly or contact the study coordinator prior to study participation.

The main data sets were obtained from Greece (*n* = 129), Cyprus (*n* = 73), Turkey (*n* = 287), Iran (*n* = 87), and the USA (*n *= 848). The country groupings are based on cultural and location similarities. For example, Greece and Cyprus are Southern European, primarily orthodox, Greek-speaking while Iran and Turkey are Central-Western Asian and Muslim countries. It should be noted that we collected data from a small sample (13 participants) from 10 other countries. We did not include this data in the subgroup analysis by region as they did not fit into the country grouping criteria. Ethical approval was provided by the University of Arizona. Additionally, the study was approved by the institutional ethics review board at each participating university before data collection. Inclusion criteria comprised adults (18 + years old), ability to read and write in the presented language of the survey (English, Iranian, Turkish, Greek) and experienced the death of a loved one. The survey was conducted anonymously via the online-platform Qualtrics. Eligible participants were informed beforehand about the study goals, risks, and benefits and gave their written consent to participate in the study. All participants were provided with debriefing forms according to the Helsinki Declaration. The completion time of the survey was approximately 40 minutes.

### 2.2. Measures

The survey was divided into 12 sections including informed consent, demographics, loss variables, scales to assess symptoms of grief and further sequelae of distress (PTSD, anxiety, depression, somatic symptom distress), and a final debriefing as well as the opportunity to submit questions to the researchers. If versions of the scales were not standardized or available in the required language, they were translated following recommended translation protocols. For example, for the Persian and Greek versions of the scales, forward translation was performed by two fluent native Greek and Persian speakers. Back translation was performed by a master translator and consensus was obtained. The Turkish translation used the five-step translation process where (1) two translators did forward translation, (2) two translators did backward translation, (3) all translators discussed the translation, (4) external individuals provided feedback, and (5) the final translation was produced. The details can be found here: https://psysciacc.org/translation-process/ (Killikelly et al., 2023).

The full list of self-report measures and all data is publicly available on the Open Science Framework (https://osf.io/tyz3u/).

#### 2.2.1. Prolonged grief symptoms

Symptoms of prolonged grief were assessed with the International Prolonged Grief Disorder Scale (IPGDS; α = .96), which has been previously validated in cross-cultural samples (Killikelly et al., 2020). The scale consists of two parts (part one is 13 items and based on the ICD-11 PGD definition, part two is the cultural supplement and includes 20 additional items that may be culturally relevant) with 33 items in total, assessing the frequency of preoccupation (e.g. ‘I am preoccupied with thoughts about the deceased or circumstances of the death.’), yearning (e.g. ‘I am longing or yearning for the deceased’) and symptoms of emotional distress and impairment (e.g. ‘I feel emotionally numb.’) over the past week in response to the death of a loved one. Answers are rated on a five-point scale (1 ‘not at all’ to 5 ‘always’) and the total score ranges between 33 to 165. A score > 72.5 was proposed as a cutoff for clinically severe symptoms of PGD (Killikelly et al., 2020). The reliability of the scale (Cronbach’s α) was .96 in the current sample. This measure was translated into Persian, Greek, and Turkish for the current study.

#### 2.2.2. Somatic symptom distress

Somatic symptom distress was assessed by the Somatic Symptom Scale (SSS-8; α = .81) (Gierk et al., 2014). The SSS-8 includes 8 symptoms derived from the Patient Health Questionnaire (PHQ-15) (e.g. stomach or bowel problems, headaches, trouble sleeping) that account for more than 90% of somatic symptoms in primary care and which are rated according to their burdensomeness in the last 7 days from 0 (‘not at all’) to 4 (‘very much’). Thus, the total score ranges from 0 to 32 with cut-off points of 4, 8, 12, and 16 representing thresholds for low, moderate, high, and very high somatic symptom burden. A cut-off ≥ 12 was proposed as a threshold for indicating a somatic symptom disorder according to DSM-5 (Toussaint et al., 2019). The reliability of the SSS-8 was high (α = .87) in the current sample. This measure was translated into Persian, Greek, and Turkish for the current study.

#### 2.2.3. Negative affect

Negative affect in terms of depressive symptoms was assessed by the Patient Health Questionnaire, depression scale (PHQ-9; α = .89) (Kroenke & Spitzer, 2002), which includes nine symptoms of depression that are rated for the last two weeks on a 4-point scale (0 = ‘not at all’ to 3 = ‘nearly every day’). Scores range between 0 to 27 points, with a recommended cut-off point for clinically significant depression ≥ 10. The reliability of the PHQ-9 was high (α = .90) in our sample. Previously validated versions of the PHQ 9 were administered in Greek (Hyphantis et al., 2011), Persian (Dadfar et al., 2018), and Turkish (Sari et al., 2016). As another aspect of negative affect, anxiety was assessed by the Generalized Anxiety Disorder Questionnaire (GAD-7; α = .92) (Spitzer et al., 2006), which includes seven symptoms of general anxiety which are rated for the last two weeks on a 4-point scale (0 = ‘not at all’ to 3 = ‘nearly every day’). Scores range from 0 to 21 points, with a proposed cut-off for clinically relevant anxiety ≥ 10. The reliability of the scale was high (α = .94) in this study. Previously validated versions of the GAD 7 were administered in Greek and Turkish (https://www.phqscreeners.com/select-screener). This measure was translated into Persian for the current study.

#### 2.2.4. Demographic and loss-related variables

Demographic characteristics included gender (male/female), age, education (high school diploma, associate degree, bachelor’s degree, master’s degree, doctorate, other, less than high school), and occupation (working/studying, unemployed/looking for work, housekeeping/care work, being retired). Loss-related variables included relationship to the deceased (first degree relative, spouse/former spouse, second degree relative, friend or, other) and cause of death (natural causes, accident, suicide, substance abuse, homicide, natural disaster).

#### 2.2.5. Statistical analyses

Ten participants aged < 18 years were excluded. Missing values in the items were replaced by the item mean and 59 cases (4.2%) with missingness > 90% per questionnaire were deleted. Possible PGD status (non-PGD/PGD) was determined using the proposed cut-off score by Killikelly et al. (2020). Chi-square (χ²) tests, independent samples *t*-tests (Welch’s *t*-test in case of variance heterogeneity), and univariate analysis of variance (ANOVA) were used to test group differences. We conducted zero-order correlations and partial correlations (controlled for negative affect) for the association between symptoms of PGD and somatic symptom distress. We detected no signs of multicollinearity or autocorrelation of residuals. Using studentized excluded residuals, 10 outliers (0.7%) were identified and regression and mediation analyses were repeated excluding these values. Since the main results of these analyses were not altered and other indices for detecting outliers (i.e. Cook’s distance and leverage values) were unremarkable, we thus report the results based on the total sample. Multicategorical predictors were dichotomized, including education (A-level vs. non-A-level), cause of death (natural vs. non-natural), and relationship to the deceased (first degree relative or spouse vs. second degree relative, friend, or other). For subgroup analyses, the residence regions of participants were grouped into USA (*n* = 826), Turkey/Iran (*n* = 320), and Cyprus/Greece (*n* = 179). To investigate predictors of somatic symptom distress, demographic characteristics (age, gender, education), loss-related variables (relationship to deceased, cause of death), PGD symptoms, and negative affect (depression, anxiety) were entered into a hierarchical multiple regression model. A three-step approach was chosen. In the first step, PGD symptoms were included as a predictor. In the second step, demographic and loss-related variables were added, and in the third step, anxiety, and depression. In this way, we were able to test if the effect of PGD symptoms on somatic symptom distress remains after controlling for covariates and also examine the particular improvements in explained variance when adding variables of negative affect. Regression analyses were also stratified by gender and region as subgroup analyses. A possible mediation of the association of PGD symptoms and somatic symptom distress by both anxiety and depression was further tested with the PROCESS macro, v3.5.3 (Hayes, 2017), using a parallel mediation model with ordinary least squares regression to estimate unstandardized coefficients, controlling for demographic and loss-related variables as covariates (95% confidence intervals, 10,000 bootstrapped samples, and heteroscedasticity consistent standard errors). Mediation was further stratified by residence region. Two cases with missings in demographic- or loss-related variables were removed from regression and mediation analyses by listwise deletion. Data analyses were performed using SPSS (v.23) with a significance level of .05 (two-sided).

### 2.3. Participant characteristics

Table 1 contains details of the participants’ characteristics. Of the 1337 participants, the mean age was 23.79 years (*SD* 8.59, range 18–82), 1018 (76.1%) were female and 876 (65.6%) had no higher education. In the US-American sample, 584 of 825 respondents (70.7%) reported a Caucasian descent, 80 (9.7%) Asian (American), 58 (7%) African (American) descent, or others (103, 12.5%). In the overall sample, most participants (78.2%) had lost a second-degree relative or friend and natural causes of death were most common (79%). Three hundred and thirteen participants (23.4%) scored above the proposed cut-off for clinically severe PGD. Possible PGD was significantly more frequent in individuals with no higher education (i.e. less than A-level) and when the deceased was a second degree relative in contrast to other relationships. No significant differences were observed for age, gender or occupation. Prevalence of possible PGD significantly differed between cultural groups and was highest among individuals from Cyprus/Greece (43.6%) compared to the USA (21.1%), and Turkey/Iran (18.4%).

**Table 1.** Sample characteristic, mean (SD) or %, (n) for the total and subsamples (possible PGD status).

Variable	Total (*N* = 1337)	Non-PGD (*n* = 1024)	PGD (*n* = 313)	Test statistics^a^
Age	23.74 (8.59)	23.64 (8.76)	24.29 (8.02)	*t*(1335) = −1.16, *p* = .245, *d* = 0.08
**Gender**				χ²(1) = 1.73, *p* = .188
Male	23.9 (319)	24.7 (253)	21.1 (66)	
Female	76.1 (1018)	75.3 (771)	78.9 (247)	
**Education (*n* = 1336)**				χ²(1) = 3.94, *p* = .047
Higher education^b^	34.4 (460)	33.0 (338)	39.1 (122)	
No higher education	65.6 (876)	67.0 (686)	60.9 (190)	
**Occupation (*n* = 1335)**				χ²(1) = 4.81, *p* = .186
Working/studying	72.1 (962)	73.2 (748)	68.4 (214)	
Unemployed/looking for work	24.9 (333)	24.2 (247)	27.5 (86)	
Housekeeping/care work	2.2 (30)	1.9 (19)	3.5 (11)	
Retired	0.7 (10)	0.8 (8)	0.6 (2)	
**Residence region (*n* = 1325)**^c^				χ²(2) = 47.45, *p* < .001
USA	62.3 (826)	78.9 (652)	21.1 (174)	
Turkey/Iran	24.2 (320)	81.6 (261)	18.4 (59)	
Cyprus/Greece	13.5 (179)	56.4 (101)	43.6 (78)	
**Loss related variables**	** **	** **	** **	** **
**Cause of death (*n* = 1336)**				χ²(4) = 9.96, *p* = .041
Natural cause	79.0 (1056)	80.7 (826)	73.5 (230)	
Accident	10.3 (137)	9.2 (94)	13.7 (43)	
Suicide	6.1 (81)	5.9 (60)	6.1 (21)	
Substance abuse	3.1 (42)	3.0 (31)	3.5 (11)	
Homicide	1.5 (20)	1.2 (12)	2.6 (8)	
**Relationship to deceased**				χ²(4) = 92.73, *p* < .001
First degree relative	16.8 (224)	12.2 (125)	31.6 (99)	
Spouse/former spouse	1.6 (22)	1.0 (10)	3.8 (12)	
Second degree relative	65.9 (881)	72.0 (737)	46.0 (144)	
Friend	12.3 (164)	11.7 (120)	14.1 (44)	
Other	3.4 (46)	3.1 (32)	4.5 (14)	
**Psychopathology**				
Symptoms of PGD (IPGDS-33)	59.45 ( 22.54)	49.17 (10.95)	93.07 (17.37)	*t*(390.65) = −42.23, *p* < .001, *d* = 3.45
Somatic symptom distress (SSS-8)	8.29 (7.19)	6.88 (6.36)	12.91 (7.79)	*t*(446.13) = −12.48, *p* < .001, *d* = 0.90
Anxiety (GAD-7)	6.49 (5.77)	5.15 (5.04)	10.86 (5.87)	*t*(460.89) = −15.54, *p* < .001, *d* = 1.09
Depression (PHQ-9)	6.72 (6.26)	5.22 (5.36)	11.62 (6.47)	*t*(450.76) = −15.91, *p* < .001, *d* = 1.13

^a^ Welch’s *t*-test reported in case of variance heterogeneity. ^b^A-levels and above. ^c^Percent per column.

## 3. Results

### 3.1. Prevalence of somatic symptom distress

**Figure 1.**
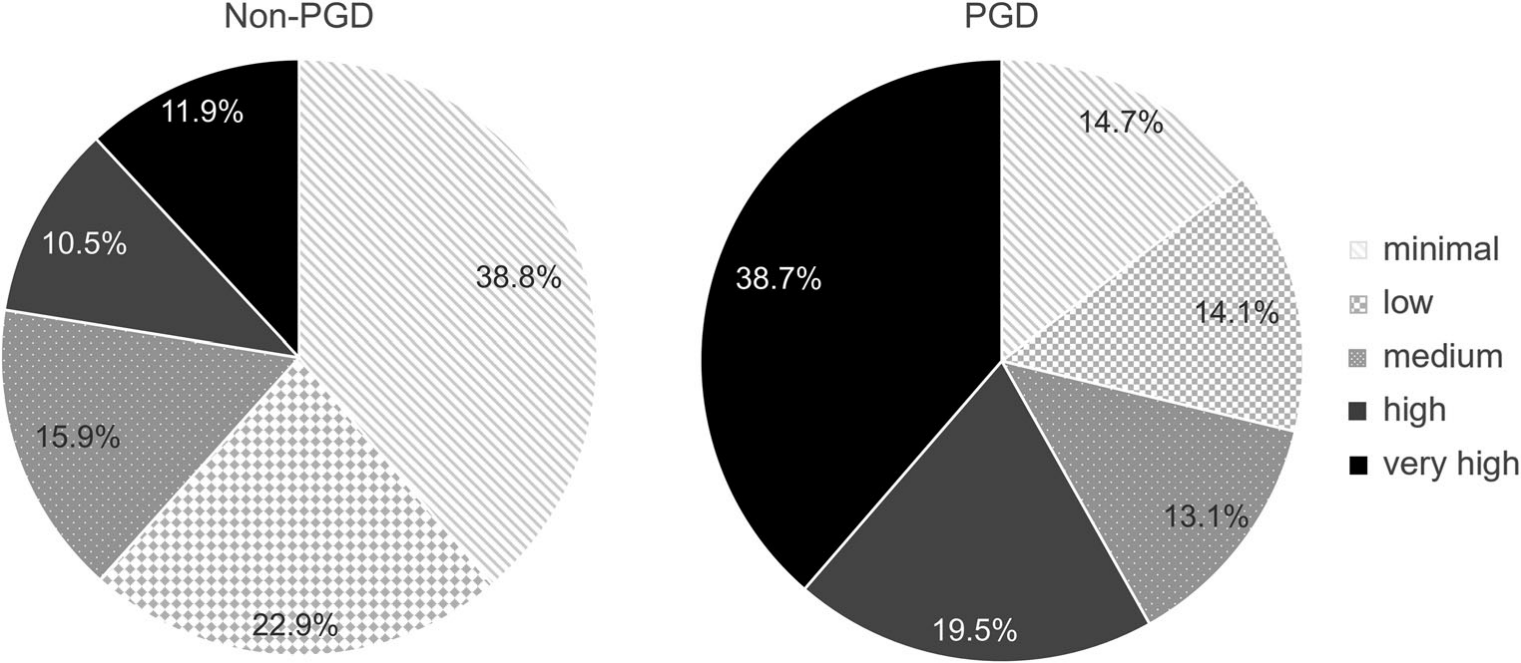
Frequency (%) of somatic symptom distress levels (assessed by the SSS-8) and probable PGD status (*N* = 1337).

In the overall sample, the mean score in the SSS-8 was 8.29 (*SD* = 7.19) and 412 participants (30.8%) showed ‘high’ or ‘very high’ levels of somatic symptom distress (see Figure 1). Average somatic symptom distress was significantly higher in female participants (Welch’s *t*(559.69) = 5.55, *p* < .001, *d* = 0.36) and significantly differed between the three regions (USA: *M* = 7.20, *SD* 6.92; Turkey/Iran: *M* = 9.37, *SD* 6.95; Cyprus/Greece: *M* = 11.32, *SD* 7.66), *F*(2,1322) = 30.39, *p* < .001, η² = 0.04. ‘High’ or ‘very high’ levels of somatic symptom distress were more frequent in the possible PGD group (58.2%), than in the non-PGD group (22.4%) χ²(4) = 161.60, *p* < .001, see Figure 1 and the average score incidated clinical relevance (≥12) in the possible PGD group (see Table 1). Mental health treatment engagement (no/yes) was not significantly associated with somatic symptom distress within the possible PGD group (*ρ *=  .069, *p* = .223).

### 3.2. Regression analyses

Zero-order correlations between symptoms of PGD and other measures of psychopathology were significantly large (*r* = .48 to .55, see Supplementary Material A), while the partial correlation of symptoms of PGD and somatic symptom distress, controlling for negative affect, was much weaker, yet significant (*r* = .09, *p* = .001). The results of the regression analyses are listed in Table 2. Symptoms of PGD remained a significant positive predictor of somatic symptom distress in all three models (model 1: *R²* = .23, model 2: *R²* = .25, model 3: *R²* = .58). In the full model, the predictive effect of PGD symptoms (*β* = 0.08, *p* < .001) was small but significant and lower than for depression (*β* = 0.52, *p* < .001) and anxiety (*β* = 0.22, *p* < .001), which formed the strongest predictors of somatic symptom distress. Only gender (*β* = 0.05, *p* = .005) was identified as another significant predictor. When analyses were stratified by gender PGD symptoms remained a significant predictor of somatic symptom distress in female individuals (*β* = 0.11, *p* < .001) but not in male participants (*β* = 0.01, *p* = .815), see Supplementary Material B.

**Table 2.** Results of the hierarchical regression predicting somatic symptom distress (*n* = 1335 participants with complete data).

Model		*B*	*SE*	β	*t*	*p*	95% CI for B	
1 (*R²* = .23)								
	PGD symptoms^a^	0.15	0.01	0.48	19.97	<.001	0.14	0.17
2 (*R²* = .25)								
	PGD symptoms	0.15	0.01	0.48	19.27	<.001	0.14	0.17
	Sex	1.86	0.40	0.11	4.61	<.001	1.07	2.65
	Age	0.01	0.03	0.01	0.22	.825	−0.04	0.05
	Education	0.68	0.43	0.04	1.60	.110	−0.15	1.51
	Cause of death	1.07	0.42	0.06	2.51	.001	0.23	1.90
	Relationship to deceased	1.33	0.50	0.07	2.64	.009	0.34	2.31
3 (*R²* = .58)								
	PGD symptoms	0.02	0.01	0.08	3.44	.001	0.01	0.04
	Sex	0.86	0.30	0.05	2.84	.005	0.27	1.46
	Age	0.02	0.02	0.02	1.01	.312	−0.02	0.06
	Education	0.11	0.32	0.01	0.35	.730	−0.51	0.73
	Cause of death	0.46	0.32	0.03	1.46	.145	−0.16	1.09
	Relationship to deceased	0.59	0.38	0.03	1.58	.115	−0.15	1.33
	Anxiety^b^	0.28	0.04	0.22	7.29	<.001	0.20	0.35
	Depression^c^	0.59	0.03	0.52	17.01	<.001	0.53	0.66

Note: PGD = Prolonged Grief Disorder. Somatic symptom distress assessed by the Somatic Symptom Scale (SSS-8). ^a^Assessed by the International Prolonged Grief Disorder Scale (IPGDS-33); ^b^assessed by the Patient Health Questionnaire, depression scale (PHQ-9); ^c^assessed by the Generalized Anxiety Disorder Questionnaire (GAD-7). Regression constant not displayed.

Furthermore, when stratified by region, PGD symptoms remained a significant predictor of somatic symptom distress in the full model in participants from USA (*β* = 0.06, *p* = .033) and from Cyprus/Greece (*β* = 0.15, *p* = .014) but not in participants from Turkey/Iran (*β* = 0.07, *p* = . 190), see Supplementary Material C.

### 3.3. Mediation analysis

**Figure 2.**
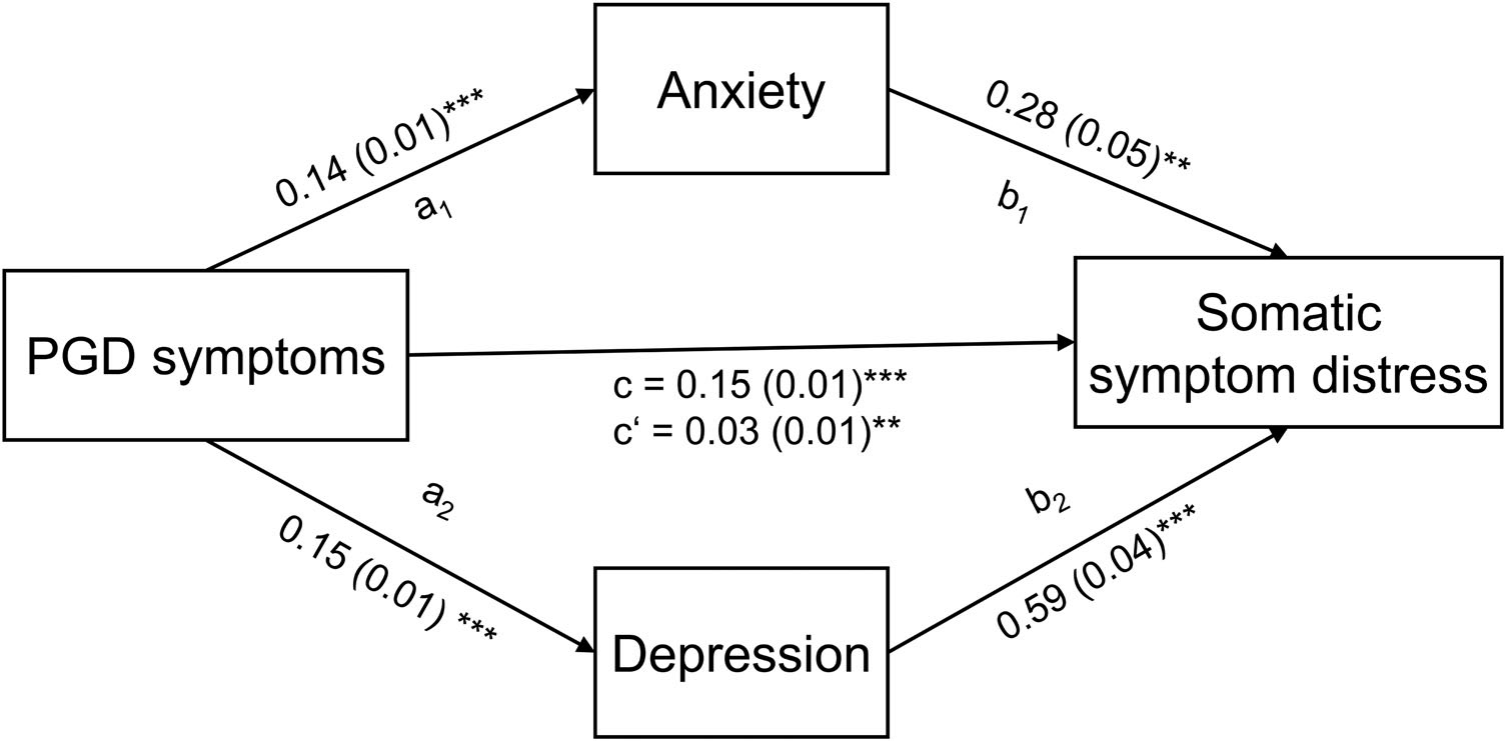
Parallel mediation model in *n* = 1335 participants with complete data. Unstandardized estimates with heteroscedasticity consistent standard errors (HC3) in brackets. *** *p* < .001, ** *p* < .01, *p < .05. PDG = Prolonged Grief Disorder.

When controlling for demographic and loss-related variables, anxiety (*R*² = .31, indirect effect_ab1_ = 0.04, 95% CI [0.03, 0.05]) and depression (*R*² = .31, indirect effect_ab2_ = 0.09, 95% CI [0.08, 0.11]) mediated the relationship between PGD symptoms and somatic symptom distress (see Figure 2). The total indirect effect explained 58% of the variance in somatic symptom distress. The direct effect of PDG on somatic symptom distress remained significant when including mediators (c = 0.03, 95% CI [0.01, 0.04], *p* = .003), indicating a partial mediation of somatic symptom distress (see Supplementary Material D for detailed results).

However, when stratified by region, this direct effect was no longer significant in participants from the USA or Cyprus/Greece (see Supplementary Material E), indicating full mediation. In participants from Cyprus/Greece, the confidence interval indicated no significant indirect effect of anxiety on somatic symptom distress (ab_1_ = 0.02 [−0.01, 0.06]), while the other results were largely comparable to the total sample.

## 4. Discussion

This is the first study to explore the relationship between somatic symptom distress and prolonged grief disorder in a large international cross-sectional survey. The main findings include an apparently high prevalence of somatic symptom distress (around 31%) and this was particularly high in females, the Cyprus/Greek sample, and the probable PGD group. Additionally, PGD symptoms predicted symptoms of somatic symptom distress while controlling for demographics, loss-related variables, or negative affect (i.e. depression, anxiety). Our findings also show that depression and anxiety partially mediate the association between PGD symptoms and somatic symptom distress.

Around two-thirds of individuals with possible PGD reported high or very high levels of somatic symptom distress in the SSS-8, which is remarkably higher than prevalences in the general population (Gierk et al., 2014), yet lower than in primary care patients (Toussaint et al., 2017) or patients with somatic symptom disorder (Toussaint et al., 2019). Compared to data from a survey of the Hungarian general population (Thege et al., 2012), somatic symptom distress was considerably higher in our study sample. Indeed, high levels of somatic symptom distress have also been found in a recent analysis of complex PTSD, another stress-related disorder (Astill Wright et al., 2021). Our findings also support a strong relationship between somatic symptom distress, depression, and anxiety symptoms, all within the context of bereavement, replicating some earlier findings (Thege et al., 2012). This is mirrored in previous studies that conclude that somatic symptoms may be common across mental disorders (Kroenke, 2003). Recently research and theory exploring ‘common processes’ underlying mental disorders has been interpreted according to a ‘systems view’ which focuses on the way individual components interact within a system or network. This ‘system’ may have different levels of interaction and explanation, for example, it has been a longstanding view of psychiatry that mental disorders originate from dysfunction at the neurobiological level (Boer et al., 2021). For example, a cross-cultural examination of somatic symptoms underlying depression in Turkish and German samples concluded that somatic symptom distress may be a core symptom at the neurobiological level (Ebert & Martus, 1994), however, empirical data to support this consistently is lacking (Boer et al., 2021). Network theory asserts that mental disorders result from the interaction of symptom networks (Borsboom, 2017). This shifts the focus from the brain level to considering symptoms in interaction with other factors, including environmental or cultural factors. Instead of suggesting that symptoms represent an underlying latent disorder for example, that PGD is the latent cause of somatic and grief-related symptoms, network theory suggests that it is the interaction between these symptoms that cause the disorder profile. The current findings indicate that there is perhaps a complex network of symptoms that interact to result in PGD disorder presentation. For example, the high prevalence of somatic symptoms followed by the strong relationship with anxiety and depression indicates that behind the PGD diagnosis are additional symptoms that may be ‘masked’ by somatic complaints (Thege et al., 2012), however, the cross-sectional nature of this study precludes causal interpretations. Anxiety and depression are often found to be comorbid with PGD (Boelen, 2013) and although they are distinct disorders they may share a common etiology after the death of a loved one. This may be missed if only considering the PGD symptom items. The picture becomes even more complex when considering the different symptom severity across cultures and including environmental factors.

There are several recent studies that have sought to examine possible gender differences in the experience and expression of grief. A recent longitudinal study found that men and women have different symptoms trajectories of grief symptoms. Men were found to have higher baseline grief symptoms that decreased overtime whereas women displayed a mounting grief reaction increasing overtime (Lundorff et al., 2020). Some researchers hypotheses that the gender differences related to bereavement can be attributed to differences in ‘amount’ not ‘kind’. As women are more likely to experience a protracted and intensifying grief response, perhaps they are more likely to experience a global impairment encompassing grief symptoms, somatic symptoms and other symptoms of psychopathology (Thompson & Bland, 2018), which is in line with our findings regarding higher levels of distress of female participants across measures and also a stronger association of symptoms of PGD and somatic symptom distress in this group, although this effect was rather small.

Indeed, we have recently confirmed different symptom network profiles for different cultural groups (Killikelly et al., 2023). Network analysis revealed that the Turkey/Iran and Greece/Cyprus groups had stronger network connections with somatic symptoms such as feeling paralyzed and having no energy. In this current study, across cultural groups, somatic symptom distress and symptoms of PGD were most prevalent in individuals from Cyprus/Greece. This subsample had a higher proportion of deceased being first degree relatives, as well as older, and higher educated participants. In this case, the network theory could explain these differences in cultural groups by including environmental factors in the network structure. Environmental or cultural factors may act as a catalyst that facilitates the change from an acute response, such as the normal grief response, to a chronic condition, PGD (Boer et al., 2021). At the individual level, some could have a vulnerability to the development of PGD based on their previous or current experience with mental health conditions such as anxiety or depression, as a dose-vulnerability model might predict (Kaysen et al., 2010). The finding that depression and anxiety partially mediate the relationship between PGD and somatic symptoms could indicate several hypothesis 1) severe symptoms of grief are more likely associated with current co-morbid anxiety and depression due to specific overlapping symptoms such as preoccupation or withdrawal, 2) High PGD symptoms may indicate impairments across several areas of psychopathology of which symptoms of PGD, anxiety, depression and somatic symptoms are some of the highest, 3) Those individuals with a history of anxiety and depression are more likely to have higher symptoms of PGD and somatic symptoms because of a kindling effect, i.e. a stronger reaction to stressful life events after previous episodes of psychopathology (Weiss et al., 2015). Future studies should examine the relationship between co-morbid disorder onset (anxiety and depression) and the trajectory of grief symptoms overtime.

### 4.1. Limitations, strengths, and future directions

In interpreting our findings, several limitations have to be considered. Firstly, the cross-sectional nature of our study prevents a causal interpretation of associations in regression analyses. To the best of our knowledge, longitudinal studies on PGD symptoms and somatic symptom distress that include pre-loss data are missing. Secondly, the artificial grouping of regions and their different size may represent further limitations, since this was based on local proximity rather than cultural aspects. Also, the categorization of clinically relevant PGD symptoms has to be considered preliminary, since more studies are needed to establish valid cut-offs for the IPGDS, particularly in terms of time since loss. Existing diagnositic manuals use two different time criteria for determining the cut off time for a diagnosis (more than 6 months, ICD-11 and 12 months DSM V-TR). In the current study we included all participants who had lost a loved one regardless of time since loss. Our main aim was to explore the relationship between severity of PGD symptoms and somatic symptoms in a general population sample. Future studies should examine the relationship between different PGD diagnostic criteria (e.g. more than 6 or 12 months since loss) and somatic symptoms in a clinical sample. Thirdly, the sample could be considered heterogeneous, e.g. regarding time since loss, and a self-selection effect for survey participation cannot be ruled out. Among the strengths of the current study are the inclusion of validated and reliable instruments, particularly for ICD-11 PGD, extending previous evidence based on a different assessment of negative affect (Thege et al., 2012). In contrast to previous studies which included mostly specific groups, cultural backgrounds, or ethnicities and were based on rather small sample sizes, we recruited a large, and cross-cultural sample. Also, we were able to perform a more fine-grained analysis by controlling for various covariates. However, additional unmeasured variables (e.g. medical conditions, somatic symptom catastrophizing, medication) could have also influenced the association of PGD symptoms and somatic symptom distress and therefore should be considered in future studies. The findings from the current study can reasonably be generalized across both WEIRD (white, educated, industrialized, rich, democratic) and non-WEIRD samples (Henrich et al., 2010). We had the opportunity to examine symptoms of PGD, depression, anxiety, and somatic symptoms of distress for the first time in different regions around the world. High somatic symptoms were found across all groups with the highest in Cyprus/Greece and lowest in USA. Further research is needed to examine these symptom relationships in a clinical sample of PGD patients.

### 4.2. Conclusion

Elevated somatic symptom distress can be observed in a substantial proportion of bereaved individuals across cultures. Our results provide support to the idea that to some extent, somatic symptom distress ‘masks’ anxiety and depression, looming in the background of grief. However, the latter also showed unique influences on somatic symptom distress, which should be explored further. A psychosomatic perspective can help to improve the understanding of the multi-faceted symptom network of PGD across cultures and encourage practitioners to consider somatic symptom distress in psychotherapeutic treatment.

## Supplementary Material

Supplemental Material

## Data Availability

The full list of self-report measures and all data is publicly available on the Open Science Framework (https://osf.io/tyz3u/).
