# Supplementary material for: Somatic symptom distress and ICD-11 prolonged grief in a large intercultural sample
Source: Eur J Psychotraumatol. 2023 Sep 28;14(2):2254584. doi: 10.1080/20008066.2023.2254584 (PMC10540649; doi:10.1080/20008066.2023.2254584)
Supplement: Supplemental Material [file ZEPT_A_2254584_SM5078.docx]

# Supplemental Material

| A | Correlation matrix |
| --- | --- |
| B | Multiple regression analysis stratified by sex |
| C | Multiple regression analysis stratified by region |
| D | Detailed results of parallel mediation analysis |
| E | Parallel mediation analysis stratified by region (Figures) |

# Supplementary Material A

Pearson correlation matrix of demographic-, loss-related-, and psychopathology variables (*n* = 1335)

|  | IPGDS-33 | SSS-8 | GAD-7 | PHQ-9 | Sex | Age | Education | Cause of death | Relationship to deceased |
| --- | --- | --- | --- | --- | --- | --- | --- | --- | --- |
| IGPDS-33 | 1.00 | 0.48 | 0.55 | 0.55 | 0.07 | 0.06 | 0.06 | 0.12 | - 0.14 |
| SSS-8 |  | 1.00 | 0.69 | 0.74 | 0.15 | 0.04 (n.s.) | 0.07 | 0.11 | -0.0003 (n.s.) |
| GAD-7 |  |  | 1.00 | 0.80 | 0.15 | 0.04 (n.s.) | 0.07 | 0.11 | -0.03 (n.s.) |
| PHQ-9 |  |  |  | 1.00 | 0.11 | 0.01 (n.s.) | 0.07 | 0.11 | -0.012 (n.s.) |
| Sex |  |  |  |  | 1.00 | 0.07 | 0.11 | 0.01 (n.s.) | -0.03 (n.s.) |
| Age |  |  |  |  |  | 1.00 | 0.53 | -0.06 | -0.30 |
| Education |  |  |  |  |  |  | 1.00 | -0.02 (n.s.) | -0.15 |
| Cause of death |  |  |  |  |  |  |  | 1.00 | 0.15 |
| Relationship to deceased |  |  |  |  |  |  |  |  | 1.00 |
| *Note.* IPGDS-33 = International Prolonged Grief Disorder Scale; SSS-8 = Somatic Symptom Scale (SSS-8). PHQ-9 = Patient Health Questionnaire, depression scale; GAD-7 = Generalized Anxiety Disorder Questionnaire; Sex = female/male; Educationn = A-level/non-A-level, Relationship to the deceased = 1st degree relative/other; Cause of death = natural/non-natural. n.s. = non-significant (all other correlations *p* < .05) | | | | | | | | | |

# Supplementary Material B

Results of hierarchical regression of somatic symptom distress stratified by sex (male: *n* = 319, female: *n* = 1016)

| Sex | | | Unstandardized Coefficients | | Standardized Coefficients | t | Sig. | 95% Confidence Interval for B | |
| --- | --- | --- | --- | --- | --- | --- | --- | --- | --- |
|  |  |  | B | SE | β |  |  | Lower Bound | Upper Bound |
| Male | 1 (R² = .15) | (Constant) | -0.49 | 0.99 |  | -0.49 | 0.622 | -2.43 | 1.45 |
|  |  | PGD symptoms | 0.12 | 0.02 | 0.39 | 7.49 | < .001 | 0.09 | 0.15 |
|  | 2 (R² = .19) | (Constant) | -0.40 | 2.81 |  | -0.14 | 0.888 | -5.93 | 5.14 |
|  |  | PGD symptoms | 0.11 | 0.02 | 0.35 | 6.66 | < .001 | 0.08 | 0.14 |
|  |  | Age | 0.02 | 0.06 | 0.02 | 0.34 | 0.735 | -0.09 | 0.13 |
|  |  | Education | 1.11 | 0.97 | 0.07 | 1.14 | 0.254 | -0.80 | 3.02 |
|  |  | Cause of death | 3.11 | 0.87 | 0.18 | 3.58 | < .001 | 1.40 | 4.83 |
|  |  | Relationship to deceased | -0.44 | 1.02 | -0.02 | -0.43 | 0.669 | -2.45 | 1.58 |
|  | 3 (R² = .52) | (Constant) | 1.27 | 2.18 |  | 0.58 | 0.560 | -3.02 | 5.56 |
|  |  | PGD symptoms | 0.00 | 0.01 | 0.01 | 0.23 | 0.815 | -0.03 | 0.03 |
|  |  | Age | 0.05 | 0.04 | 0.05 | 1.11 | 0.270 | -0.04 | 0.13 |
|  |  | Education | -0.25 | 0.76 | -0.02 | -0.32 | 0.746 | -1.73 | 1.24 |
|  |  | Cause of death | 0.97 | 0.69 | 0.06 | 1.40 | 0.162 | -0.39 | 2.32 |
|  |  | Relationship to deceased | -0.49 | 0.79 | -0.03 | -0.62 | 0.538 | -2.05 | 1.07 |
|  |  | Anxiety | 0.45 | 0.09 | 0.34 | 5.23 | < .001 | 0.28 | 0.62 |
|  |  | Depression | 0.44 | 0.08 | 0.39 | 5.75 | < .001 | 0.29 | 0.59 |
| Female | 1 (R² = .25) | (Constant) | -0.68 | 0.56 |  | -1.23 | 0.220 | -1.77 | 0.41 |
|  |  | PGD symptoms | 0.16 | 0.01 | 0.50 | 18.42 | < .001 | 0.14 | 0.18 |
|  | 2 (R² = .26) | (Constant) | -4.91 | 1.66 |  | -2.96 | 0.003 | -8.17 | -1.66 |
|  |  | PGD symptoms | 0.17 | 0.01 | 0.53 | 18.42 | < .001 | 0.15 | 0.18 |
|  |  | Age | 0.003 | 0.03 | 0.00 | 0.10 | 0.923 | -0.05 | 0.06 |
|  |  | Education | 0.52 | 0.47 | 0.03 | 1.10 | 0.271 | -0.41 | 1.44 |
|  |  | Cause of death | 0.44 | 0.48 | 0.02 | 0.91 | 0.363 | -0.51 | 1.39 |
|  |  | Relationship to deceased | 1.87 | 0.58 | 0.10 | 3.23 | 0.001 | 0.73 | 3.00 |
|  | 3 (R² = .59) | (Constant) | -1.26 | 1.24 |  | -1.02 | 0.310 | -3.68 | 1.17 |
|  |  | PGD symptoms | 0.03 | 0.01 | 0.10 | 4.03 | < .001 | 0.02 | 0.05 |
|  |  | Age | 0.01 | 0.02 | 0.02 | 0.65 | 0.515 | -0.03 | 0.05 |
|  |  | Education | 0.16 | 0.35 | 0.01 | 0.46 | 0.643 | -0.52 | 0.85 |
|  |  | Cause of death | 0.32 | 0.36 | 0.02 | 0.89 | 0.372 | -0.38 | 1.02 |
|  |  | Relationship to deceased | 0.88 | 0.43 | 0.05 | 2.06 | 0.039 | 0.04 | 1.73 |
|  |  | Anxiety | 0.23 | 0.04 | 0.18 | 5.33 | < .001 | 0.14 | 0.31 |
|  |  | Depression | 0.64 | 0.04 | 0.55 | 16.21 | < .001 | 0.56 | 0.71 |
| *Note.* DV: Somatic symptom distress (assessed by the Somatic Symptom Scale, SSS-8). IVs: PGD = Prolonged Grief Disorder, assessed by the International Prolonged Grief Disorder Scale (IPGDS-33); Education = A-level/non-A-level; Cause of death = natural/non-natural; Relationship to the deceased = 1st degree relative/other; Depression = Patient Health Questionnaire, depression scale (PHQ-9); Anxiety = Generalized Anxiety Disorder Questionnaire (GAD-7). | | | | | | | | | |

# Supplementary Material C

Results of hierarchical regression of somatic symptom distress stratified by residence region (USA: *n* = 825; Cyprus/Greece: *n* = 178; Turkey/Iran: *n* = 320)

| Residence region | | | Unstandardized Coefficients | | Standardized Coefficients | *t* | *Sig.* | 95% Confidence Interval for B | |
| --- | --- | --- | --- | --- | --- | --- | --- | --- | --- |
|  |  |  | *B* | *SE* | β |  |  | Lower Bound | Upper Bound |
| USA | 1 (R² = .22) | (Constant) | -1.13 | 0.59 |  | -1.90 | 0.058 | -2.29 | 0.04 |
|  |  | PGD symptoms | 0.14 | 0.01 | 0.46 | 15.03 | < .001 | 0.12 | 0.16 |
|  | 2 (R² = .25) | (Constant) | -6.26 | 2.03 |  | -3.09 | 0.002 | -10.24 | -2.28 |
|  |  | PGD symptoms | 0.14 | 0.01 | 0.45 | 14.01 | < .001 | 0.12 | 0.16 |
|  |  | Sex | 1.84 | 0.46 | 0.12 | 3.99 | < .001 | 0.94 | 2.75 |
|  |  | Age | -0.004 | 0.03 | 0.00 | -0.12 | 0.906 | -0.07 | 0.06 |
|  |  | Education | 0.11 | 0.68 | 0.01 | 0.15 | 0.877 | -1.24 | 1.45 |
|  |  | Cause of death | 1.80 | 0.50 | 0.11 | 3.58 | 0.000 | 0.81 | 2.78 |
|  |  | Relationship to deceased | 1.02 | 0.68 | 0.05 | 1.50 | 0.135 | -0.32 | 2.35 |
|  | 3 (R² = .58) | (Constant) | -2.49 | 1.51 |  | -1.65 | 0.100 | -5.46 | 0.48 |
|  |  | PGD symptoms | 0.02 | 0.01 | 0.06 | 2.13 | 0.033 | 0.00 | 0.04 |
|  |  | Sex | 0.96 | 0.34 | 0.06 | 2.79 | 0.005 | 0.29 | 1.64 |
|  |  | Age | 0.03 | 0.02 | 0.03 | 1.15 | 0.251 | -0.02 | 0.07 |
|  |  | Education | -0.58 | 0.51 | -0.03 | -1.13 | 0.258 | -1.58 | 0.42 |
|  |  | Cause of death | 0.49 | 0.38 | 0.03 | 1.30 | 0.193 | -0.25 | 1.23 |
|  |  | Relationship to deceased | 0.69 | 0.50 | 0.04 | 1.36 | 0.175 | -0.30 | 1.68 |
|  |  | Anxiety | 0.26 | 0.05 | 0.22 | 5.74 | < .001 | 0.17 | 0.35 |
|  |  | Depression | 0.60 | 0.04 | 0.53 | 13.94 | < .001 | 0.52 | 0.69 |
| Cyprus/  Greece | 1 (R² = .27) | (Constant) | 0.02 | 1.48 |  | 0.02 | 0.987 | -2.91 | 2.95 |
|  |  | PGD symptoms | 0.16 | 0.02 | 0.52 | 8.09 | < .001 | 0.12 | 0.20 |
|  | 2 (R² = .31) | (Constant) | 0.83 | 5.02 |  | 0.17 | 0.869 | -9.07 | 10.73 |
|  |  | PGD symptoms | 0.17 | 0.02 | 0.54 | 8.32 | 0.000 | 0.13 | 0.21 |
|  |  | Sex | -1.75 | 2.16 | -0.05 | -0.81 | 0.419 | -6.03 | 2.52 |
|  |  | Age | 0.00 | 0.06 | 0.00 | -0.02 | 0.986 | -0.12 | 0.12 |
|  |  | Education | -1.62 | 1.15 | -0.10 | -1.41 | 0.161 | -3.88 | 0.65 |
|  |  | Cause of death | -1.33 | 1.52 | -0.06 | -0.88 | 0.380 | -4.33 | 1.66 |
|  |  | Relationship to deceased | 2.24 | 1.10 | 0.14 | 2.03 | 0.044 | 0.06 | 4.42 |
|  | 3 (R² = .60) | (Constant) | 1.62 | 3.84 |  | 0.42 | 0.674 | -5.97 | 9.21 |
|  |  | PGD symptoms | 0.05 | 0.02 | 0.15 | 2.48 | 0.014 | 0.01 | 0.08 |
|  |  | Sex | -1.03 | 1.66 | -0.03 | -0.62 | 0.534 | -4.31 | 2.24 |
|  |  | Age | 0.02 | 0.05 | 0.02 | 0.42 | 0.678 | -0.07 | 0.11 |
|  |  | Education | -1.46 | 0.88 | -0.09 | -1.66 | 0.099 | -3.19 | 0.28 |
|  |  | Cause of death | 0.51 | 1.18 | 0.02 | 0.44 | 0.663 | -1.81 | 2.83 |
|  |  | Relationship to deceased | 0.89 | 0.86 | 0.06 | 1.04 | 0.301 | -0.80 | 2.58 |
|  |  | Anxiety | 0.18 | 0.11 | 0.14 | 1.68 | 0.095 | -0.03 | 0.39 |
|  |  | Depression | 0.64 | 0.10 | 0.55 | 6.24 | < .001 | 0.44 | 0.85 |
| Turkey/  Iran | 1 (R² = .21) | (Constant) | 0.02 | 1.07 |  | 0.02 | 0.988 | -2.08 | 2.12 |
|  |  | PGD symptoms | 0.17 | 0.02 | 0.46 | 9.26 | < .001 | 0.13 | 0.20 |
|  | 2 (R² = .24) | (Constant) | -2.52 | 3.70 |  | -0.68 | 0.496 | -9.80 | 4.76 |
|  |  | PGD symptoms | 0.17 | 0.02 | 0.49 | 8.94 | < .001 | 0.14 | 0.21 |
|  |  | Sex | 0.37 | 0.90 | 0.02 | 0.41 | 0.683 | -1.41 | 2.14 |
|  |  | Age | -0.08 | 0.06 | -0.07 | -1.23 | 0.220 | -0.21 | 0.05 |
|  |  | Education | -0.79 | 0.78 | -0.06 | -1.02 | 0.307 | -2.32 | 0.73 |
|  |  | Cause of death | 1.31 | 0.92 | 0.07 | 1.41 | 0.159 | -0.51 | 3.12 |
|  |  | Relationship to deceased | 1.94 | 1.08 | 0.10 | 1.80 | 0.072 | -0.18 | 4.06 |
|  | 3 (R² = .52) | (Constant) | 1.67 | 3.00 |  | 0.56 | 0.579 | -4.24 | 7.57 |
|  |  | PGD symptoms | 0.03 | 0.02 | 0.07 | 1.31 | 0.190 | -0.01 | 0.06 |
|  |  | Sex | 0.08 | 0.72 | 0.00 | 0.11 | 0.911 | -1.34 | 1.51 |
|  |  | Age | -0.04 | 0.05 | -0.04 | -0.85 | 0.395 | -0.15 | 0.06 |
|  |  | Education | 0.40 | 0.63 | 0.03 | 0.64 | 0.522 | -0.83 | 1.63 |
|  |  | Cause of death | 1.31 | 0.74 | 0.07 | 1.76 | 0.079 | -0.15 | 2.77 |
|  |  | Relationship to deceased | 0.30 | 0.87 | 0.02 | 0.35 | 0.727 | -1.41 | 2.02 |
|  |  | Anxiety | 0.41 | 0.10 | 0.29 | 4.08 | < .001 | 0.21 | 0.60 |
|  |  | Depression | 0.47 | 0.08 | 0.41 | 5.91 | < .001 | 0.31 | 0.62 |
| *Note.* DV: Somatic symptom distress (assessed by the Somatic Symptom Scale, SSS-8). IVs: PGD = Prolonged Grief Disorder, assessed by the International Prolonged Grief Disorder Scale (IPGDS-33); Education = A-level/non-A-level; Cause of death = natural/non-natural; Relationship to the deceased = 1st degree relative/other; Depression = Patient Health Questionnaire, depression scale (PHQ-9); Anxiety = Generalized Anxiety Disorder Questionnaire (GAD-7). | | | | | | | | | |

# Supplementary Material D

Results of parallel mediation analysis using the Process Macro in SPSS

Run MATRIX procedure:

**************** PROCESS Procedure for SPSS Version 3.5.3 ****************

Written by Andrew F. Hayes, Ph.D. www.afhayes.com

Documentation available in Hayes (2018). www.guilford.com/p/hayes3

**************************************************************************

Model : 4

Y : SSS8

X : IGPDS33

M1 : GAD7

M2 : PHQ7

Covariates:

Sex Age Education Cause of Death [CauseDeath] Relationship [Relationship to deceased]

Sample Size: 1335

**************************************************************************

OUTCOME VARIABLE:

GAD7

Model Summary

R R-sq MSE F(HC3) df1 df2 p

,559 ,313 23,020 97,085 6,000 1328,000 ,000

Model

coeff se(HC3) t p LLCI ULCI

constant -5,889 1,195 -4,930 ,000 -8,232 -3,545

IGPDS33 ,139 ,006 21,902 ,000 ,127 ,152

Sex 1,477 ,301 4,902 ,000 ,886 2,068

Age ,000 ,019 ,023 ,982 -,037 ,038

Education ,418 ,326 1,282 ,200 -,222 1,058

CauseDeath ,615 ,336 1,832 ,067 -,044 1,273

Relation ,673 ,392 1,715 ,087 -,097 1,442

Standardized coefficients

coeff

IGPDS33 ,543

Sex ,109

Age ,001

EDUCATIONN ,034

CauseDeath ,043

Relation ,045

**************************************************************************

OUTCOME VARIABLE:

PHQ7

Model Summary

R R-sq MSE F(HC3) df1 df2 p

,556 ,310 27,177 94,495 6,000 1328,000 ,000

Model

coeff se(HC3) t p LLCI ULCI

constant -5,649 1,282 -4,407 ,000 -8,164 -3,135

IGPDS33 ,153 ,007 20,809 ,000 ,139 ,167

Sex ,986 ,338 2,914 ,004 ,322 1,649

Age -,023 ,018 -1,237 ,216 -,059 ,013

Education ,765 ,359 2,129 ,033 ,060 1,470

CauseDeath ,730 ,372 1,962 ,050 ,000 1,461

Relation ,921 ,415 2,218 ,027 ,107 1,736

Standardized coefficients

coeff

IGPDS33 ,551

Sex ,067

Age -,031

EDUCATIONN ,058

CauseDeath ,048

Relation ,057

**************************************************************************

OUTCOME VARIABLE:

SSS8

Model Summary

R R-sq MSE F(HC3) df1 df2 p

,763 ,583 21,679 199,056 8,000 1326,000 ,000

Model

coeff se(HC3) t p LLCI ULCI

constant -2,151 1,242 -1,732 ,083 -4,587 ,285

IGPDS33 ,025 ,008 3,018 ,003 ,009 ,041

GAD7 ,277 ,047 5,887 ,000 ,184 ,369

PHQ7 ,594 ,042 14,228 ,000 ,512 ,676

Sex ,863 ,316 2,731 ,006 ,243 1,483

Age ,019 ,018 1,059 ,290 -,016 ,054

Edcucation ,110 ,335 ,328 ,743 -,548 ,768

CauseDeath ,463 ,333 1,393 ,164 -,189 1,116

Relation ,594 ,396 1,500 ,134 -,183 1,370

Standardized coefficients

coeff

IGPDS33 ,078

GAD7 ,222

PHQ7 ,517

Sex ,051

Age ,023

EDUCATIONN ,007

CauseDeath ,026

Relation ,032

Test(s) of X by M interaction:

F(HC3) df1 df2 p

M1*X ,096 1,000 1325,000 ,757

M2*X ,160 1,000 1325,000 ,689

************************** TOTAL EFFECT MODEL ****************************

OUTCOME VARIABLE:

SSS8

Model Summary

R R-sq MSE F(HC3) df1 df2 p

,501 ,251 38,842 72,741 6,000 1328,000 ,000

Model

coeff se(HC3) t p LLCI ULCI

constant -7,135 1,563 -4,565 ,000 -10,201 -4,069

IGPDS33 ,154 ,009 18,010 ,000 ,137 ,171

Sex 1,857 ,409 4,539 ,000 1,054 2,660

Age ,006 ,025 ,221 ,825 -,044 ,055

Education ,680 ,445 1,527 ,127 -,194 1,553

CauseDeath 1,067 ,433 2,465 ,014 ,218 1,916

Relation 1,327 ,500 2,656 ,008 ,347 2,307

Standardized coefficients

coeff

IGPDS33 ,483

Sex ,110

Age ,007

Education ,045

CauseDeath ,060

Relation ,071

************** TOTAL, DIRECT, AND INDIRECT EFFECTS OF X ON Y **************

Total effect of X on Y

Effect se(HC3) t p LLCI ULCI c_ps c_cs

,154 ,009 18,010 ,000 ,137 ,171 ,021 ,483

Direct effect of X on Y

Effect se(HC3) t p LLCI ULCI c'_ps c'_cs

,025 ,008 3,018 ,003 ,009 ,041 ,003 ,078

Indirect effect(s) of X on Y:

Effect BootSE BootLLCI BootULCI

TOTAL ,129 ,007 ,115 ,144

GAD7 ,039 ,007 ,026 ,052

PHQ7 ,091 ,007 ,077 ,106

Partially standardized indirect effect(s) of X on Y:

Effect BootSE BootLLCI BootULCI

TOTAL ,018 ,001 ,016 ,020

GAD7 ,005 ,001 ,004 ,007

PHQ7 ,013 ,001 ,011 ,015

Completely standardized indirect effect(s) of X on Y:

Effect BootSE BootLLCI BootULCI

TOTAL ,405 ,022 ,364 ,448

GAD7 ,121 ,021 ,080 ,163

PHQ7 ,285 ,023 ,241 ,331

*********** BOOTSTRAP RESULTS FOR REGRESSION MODEL PARAMETERS ************

OUTCOME VARIABLE:

GAD7

Coeff BootMean BootSE BootLLCI BootULCI

constant -5,889 -5,913 1,186 -8,186 -3,598

IGPDS33 ,139 ,139 ,006 ,127 ,151

Sex 1,477 1,475 ,300 ,880 2,064

Age ,000 ,001 ,019 -,034 ,039

Education ,418 ,411 ,325 -,245 1,035

CauseDeath ,615 ,619 ,334 -,037 1,264

Relation ,673 ,681 ,392 -,101 1,438

----------

OUTCOME VARIABLE:

PHQ7

Coeff BootMean BootSE BootLLCI BootULCI

constant -5,649 -5,660 1,270 -8,108 -3,148

IGPDS33 ,153 ,153 ,007 ,138 ,167

Sex ,986 ,983 ,336 ,329 1,641

Age -,023 -,022 ,018 -,057 ,015

Education ,765 ,758 ,357 ,055 1,446

CauseDeath ,730 ,740 ,369 ,034 1,466

Relation ,921 ,929 ,412 ,107 1,721

----------

OUTCOME VARIABLE:

SSS8

Coeff BootMean BootSE BootLLCI BootULCI

constant -2,151 -2,150 1,219 -4,508 ,210

IGPDS33 ,025 ,025 ,008 ,009 ,041

GAD7 ,277 ,277 ,046 ,187 ,368

PHQ7 ,594 ,594 ,041 ,514 ,676

Sex ,863 ,862 ,315 ,237 1,476

Age ,019 ,019 ,018 -,015 ,054

Education ,110 ,114 ,335 -,541 ,767

CauseDeath ,463 ,462 ,331 -,190 1,115

Relation ,594 ,594 ,391 -,182 1,381

*********************** ANALYSIS NOTES AND ERRORS ************************

Level of confidence for all confidence intervals in output:

95,0000

Number of bootstrap samples for percentile bootstrap confidence intervals:

10000

NOTE: A heteroscedasticity consistent standard error and covariance matrix estimator was used.

# Supplementary Material E

Results of parallel mediation analysis stratified by residence region (E1: USA, E2: Turkey/Iran, E3: Cyprus/ Greece).

b_2_

b_1_

a_2_

a_1_

PGD symptoms

Somatic symptom distress
Indirect effect ab: R² = .58

Anxiety
R² = .31

Depression
R² = .32

c = 0.14 (0.01)***

c‘ = 0.03 (0.01)**

0.13 (0.01)***

0.14 (0.01) ***

0.26 (0.06)***

0.60 (0.05)***

c’ = 0.02 (0.01), *p* = .061

c‘ = 0.03 (0.01)**sad

**Figure E1**

Parallel mediation model in *n* = 825 participants from USA with complete data.

Unstandardized estimates with heteroscedasticity consistent standard errors in brackets and R² of direct and indirect effects. Indirect effects: ab_1_ = 0.03 [0.02, 0.05]; ab_2_ = 0.09 [0.07, 0.10]. *** *p* < .001, ** *p* < .01, **p* < .05. PDG = Prolonged Grief Disorder.

b_2_

b_1_

a_2_

a_1_

b_2_

a_2_

b_1_

a_1_

PGD symptoms

Somatic symptom distress
Indirect effect ab: R² = .52

Anxiety
R² = .37

Depression
R² = .33

c = 0.18 (0.02)***

c‘ = 0.03 (0.01)**

0.16 (0.01)***

0.18 (0.02) ***

0.41 (0.11)***

0.47 (0.10)***

c’ = 0.03 (0.02), *p* = .243

c‘ = 0.03 (0.01)**

**Figure E2**

Parallel mediation model in *n* = 320 participants from Turkey/Iran with complete data.

Unstandardized estimates with heteroscedasticity consistent standard errors in brackets and R² of direct and indirect effects. Indirect effects: ab_1_ = 0.07 [0.03, 0.10]; ab_2_ = 0.08 [0.05, 0.12]. *** *p* < .001, ** *p* < .01, **p* < .05. PDG = Prolonged Grief Disorder.

b_2_

a_2_

b_1_

a_1_

PGD symptoms

Somatic symptom distress
Indirect effect ab: R² = .60

Anxiety
R² = .28

Depression
R² = .35

c = 0.17 (0.02)***

c‘ = 0.03 (0.01)**

0.12 (0.02)***

0.15 (0.02) ***

0.18 (0.13), *p* = .171

0.64 (0.12)***

c’ = 0.05 (0.02)*

c‘ = 0.03 (0.01)**

**Figure E3**

Parallel mediation model in *n* = 178 participants from Cyprus/Greece with complete data.

Unstandardized estimates with heteroscedasticity consistent standard errors in brackets and R² of direct and indirect effects. Indirect effects: ab_1_ = 0.02 [-0.01, 0.06]; ab_2_ = 0.10 [0.06, 0.14]. *** *p* < .001, ** *p* < .01, **p* < .05. PDG = Prolonged Grief Disorder.
